# Haploinsufficiency and triploinsensitivity of the same 6p25.1p24.3 region in a family

**DOI:** 10.1186/s12920-015-0113-1

**Published:** 2015-07-15

**Authors:** Zhongxia Qi, Linda Jo Bone Jeng, Anne Slavotinek, Jingwei Yu

**Affiliations:** Department of Laboratory Medicine, University of California San Francisco, San Francisco, CA 94107 USA; Departments of Medicine, Pediatrics and Pathology, Program for Personalized and Genomic Medicine, University of Maryland School of Medicine, Baltimore, MD 21201 USA; Department of Pediatrics, University of California San Francisco, San Francisco, CA 94143 USA

**Keywords:** Deletion of 6p25.1p24.3, Haploinsufficiency, Duplication of 6p25.1p24.3, Triploinsensitivity, Unequal sister chromatid exchange, Mosaicism

## Abstract

**Background:**

Chromosome 6pter-p24 deletion syndrome (OMIM #612582) is a recognized chromosomal disorder. Most of the individuals with this syndrome carry a terminal deletion of the short arm of chromosome 6 (6p) with a breakpoint within the 6p25.3p23 region. An approximately 2.1 Mb terminal region has been reported to be responsible for some major features of the syndrome. The phenotypic contributions of other deleted regions are unknown. Interstitial deletions of the region are uncommon, and reciprocal interstitial duplication in this region is extremely rare.

**Case presentation:**

We present a family carrying an interstitial deletion and its reciprocal duplication within the 6p25.1p24.3 region. The deletion is 5.6 Mb in size and was detected by array comparative genomic hybridization (aCGH) in a 26-month-old female proband who presented speech delay and mild growth delay, bilateral conductive hearing loss and dysmorphic features. Array CGH studies of her family members detected an apparently mosaic deletion of the same region in the proband’s mildly affected mother, but a reciprocal interstitial duplication in her phenotypically normal brother. Further chromosomal and fluorescence in situ hybridization (FISH) analyses revealed that instead of a simple mosaic deletion of 6p25.1p24.3, the mother actually carries three cell populations in her peripheral blood, including a deletion (~70 %), a duplication (~8 %) and a normal (~22 %) populations. Therefore, both the deletion and duplication seen in the siblings were apparently inherited from the mother.

**Conclusions:**

Interstitial deletion within the 6p25.1p24.3 region and its reciprocal duplication may co-exist in the same individual and/or family due to mitotic unequal sister chromatid exchange. While the deletion causes phenotypes reportedly associated with the chromosome 6pter-p24 deletion syndrome, the reciprocal duplication may have no or minimal phenotypic effect, suggesting possible triploinsensitivity of the same region. In addition, the cells with the duplication may compensate the phenotypic effect of the cells with the deletion in the same individual as implied by the maternal karyotype and her mild phenotype. Chromosomal and FISH analyses are essential to verify abnormal cytogenomic array findings.

## Background

Deletion of the distal part of 6p is a clinically recognized syndrome (chromosome 6pter-p24 deletion syndrome, OMIM #612582) characterized by developmental delay/mental retardation, language impairment, Dandy-Walker malformation, conductive hearing loss, anomalies of the anterior chamber of eyes, cardiac abnormalities, and craniofacial dysmorphism [10]. More than 30 deletion cases have been reported and the vast majority of them involve terminal deletions (0.3 - 14 Mb in size) with breakpoints within the 6p25.3p23 region [11, 12, 17, 21, 24, 25]. Only four interstitial deletions within this region have been reported [7, 14, 21]. An approximately 2.1 Mb commonly deleted terminal region was identified, which is possibly responsible for some of the major features of the syndrome [1, 10]. However, the phenotypic contributions of other deleted regions are unknown. On the other hand, duplications of the 6p25.3p23 region are extremely rare. There are only two cases with eye and cerebellar abnormalities reported in literature [29, 34].

We present here the phenotypic and genomic findings in a family with an affected female proband who carries an interstitial deletion within the 6p25.1p24.3 region, her phenotypically unaffected brother with a reciprocal interstitial duplication of the same region, and their mosaic carrier mother who carries three cell populations with the deletion, the duplication and normal cells, respectively. This rare family provided an excellent opportunity to investigate the genomic etiology and genotype-phenotype correlation of the chromosome 6pter-p24 deletion syndrome.

## Case presentation

### Proband

The female proband was born to a 33-year-old G2P2 mother at 37-weeks gestation by spontaneous vaginal delivery without complications. Pregnancy was unremarkable except for possible clubfoot noted on ultrasound scan. Birth weight was 2,440 g (20th centile), length 45.5 cm (10th centile), and head circumference 31 cm (10th centile). A hemangioma on the neck was diagnosed at one week after birth. A small patent foramen ovale (PFO, 3 mm x 4 mm) was found at 12 months of age and has been followed without surgical intervention. The proband was referred for clinical genetic evaluation at the age of 26 months for dysmorphic features, speech delay and mild growth delay. She walked at 15–16 months and her fine motor skills were age appropriate. She had four-five words at two years of age. When last reviewed at three years nine months of age, she was able to pronounce words with three syllables and had more than one hundred words. She understood multistep commands and exhibited age-appropriate behavior.

She had mild to slight conductive hearing loss at 500–4000 Hz with a notch or normal hearing at 2000 Hz and she used bilateral hearing aids from age two years 11 months until three years two months, when her 10–15 dB loss had improved. Her teeth were late to erupt and she was missing three primary teeth.

**Fig. 1:**
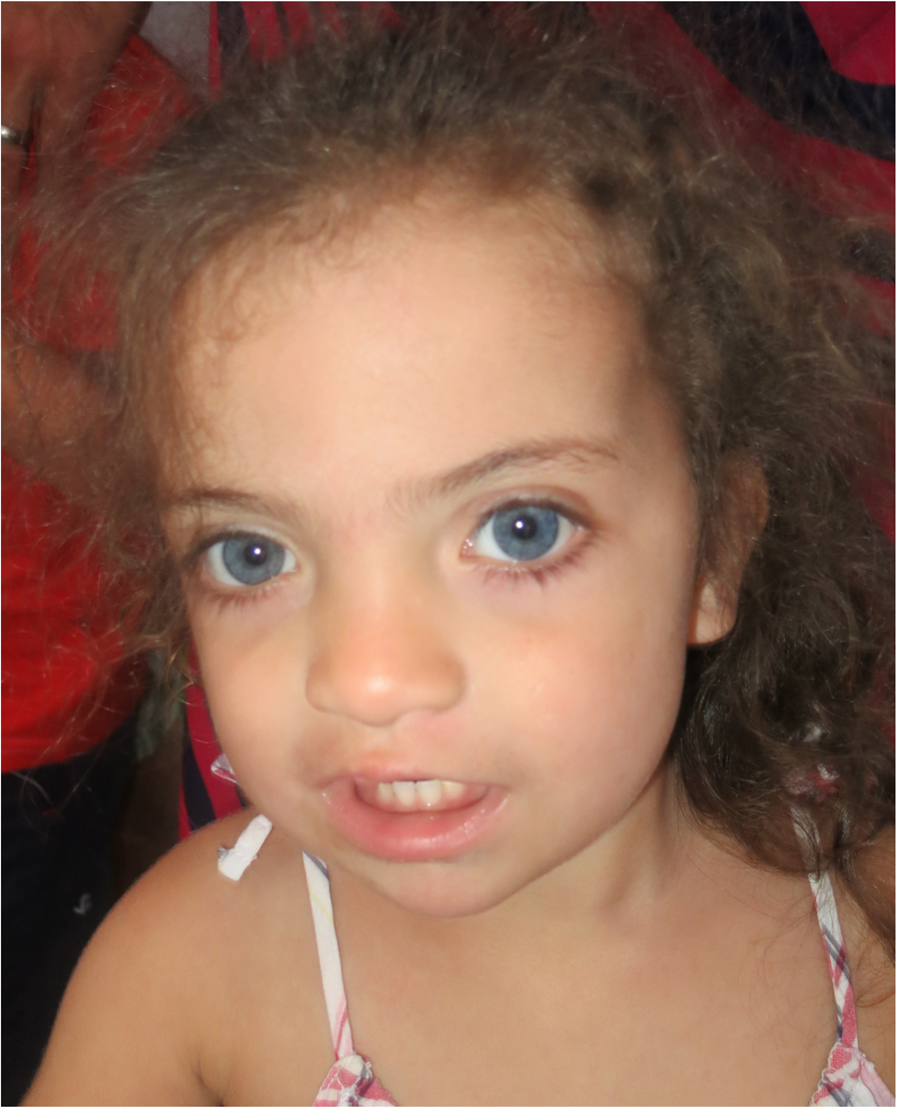
Facial photograph of the proband at the age of thee years and nine months. The picture shows high anterior hairline with sparse frontal hair, synophrys, upslanting palpebral fissures with hypertelorism and a broad nasal bridge and a short philtrum

At thee years and nine months of age, height was 90 cm (4rd centile), weight was 13.34 kg (16th centile) and occipitofrontal circumference was 48 cm (19th centile). She showed mild dysmorphic features, including sparse frontal hair with a high anterior hairline, hypertelorism with an interpupillary distance (IPD) measuring 5.8 cm (>97th centile), synophrys, a preauricular pit on the left side, short philtrum with a short columella, downturned corners of the mouth, and small, widely spaced teeth (Fig. 1). She had a resolving hemangioma on the neck that measured five cm, pectus excavatum and a small, reducible umbilical hernia. Her fingers were small with mild fifth finger clinodactyly, but measurements did not show brachydactyly. The second toe overlapped the third toe on right foot.

### Proband’s brother

The proband’s brother was delivered at 39-weeks gestation to the same mother (30 years old, G1P1) without complications. His birth weight, length, and head circumference were 3,650 g, 49.0 cm, and 35 cm, respectively, and all were within the normal range. At age of 5 years, his growth and development were appropriate for age. He had small epicanthic folds and mild clinodactyly of the fifth fingers and toes with mildly small fifth toes, but there were no other findings.

### Proband’s mother

The proband’s mother is a 35-year old and typically developed female. She had an embolic stroke at age of 26 years. Investigations with an echocardiogram showed a PFO with an atrial septal aneurysm and the PFO was closed using a transcatheter approach. Her hypercoagulability workup was negative. She had dyslipidemia with a slightly elevated lipoprotein level. Her family history was unremarkable for cardiac disease.

The proband’s father is a normal healthy male.

## Methods

### Array CGH analysis

Genomic DNA was extracted from peripheral blood using QIAGEN EZ1 kit (QIAGEN). Array CGH was performed using a custom 180 k oligonucleotide-based microarray with an International Standards for Cytogenomic Arrays Consortium V1 Clinical Design complying with the human genome build GRCh37/hg19 (Illumina). Array CGH was set up according to manufacturer’s instruction and the data were analyzed using BlueFuse Multi software (Illumina) and the UCSC genome browser (http://genome.ucsc.edu).

### Conventional cytogenetic analysis

Peripheral blood cells were cultured, harvested and banded according to the standard cytogenetic methods [3]. GTG-banded chromosomes were analyzed and imaged using the CytoVision system (Leica Microsystems). Chromosomal abnormalities were described with an International System for Human Cytogenetic Nomenclature 2013 (ISCN 2013) [28].

### FISH

**Fig. 2:**
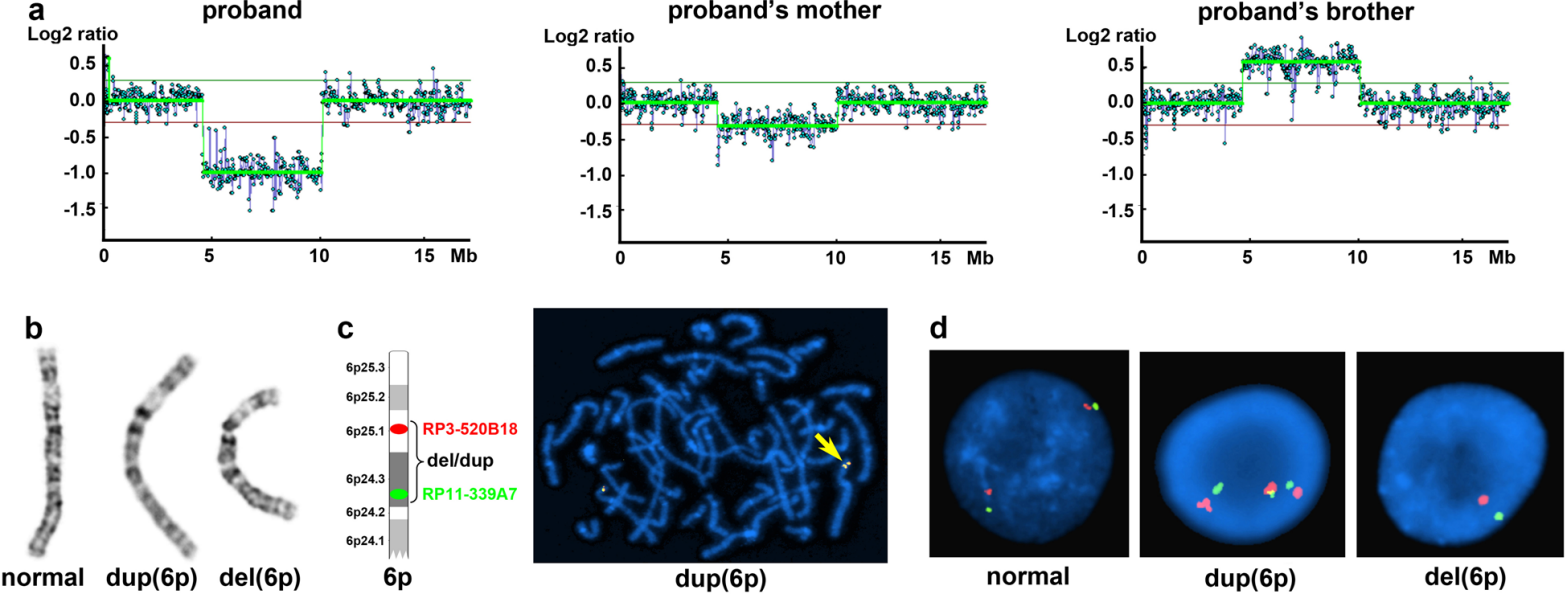
Array CGH and FISH analyses of the family. **a**. Array CGH detected an interstitial deletion within 6p25.1p24.3 in the proband, a mosaic deletion in her mother, and an interstitial duplication of the same region in her brother. **b**. Chromosomal analysis revealed normal, duplicated and deleted chromosome 6 s in the mother. **c**. Metaphase FISH showed a duplication signal pattern in a metaphase cell from the mother using two BAC FISH probes RP3-520B18 and RP11-339A7 (arrow indicates the duplication of 6p). **d**. Interphase FISH demonstrated normal, duplication, and deletion signal patterns of 6p in the mother

FISH was performed to further confirm the aCGH and chromosomal findings. Two directly labeled bacteria artificial chromosome (BAC) clones (Illumina), RP11-339A7 (6p24.3, labeled with green fluorochrome) and RP3-520B18 (6p25.1, labeled with red fluorochrome) were used as FISH probes (Fig. 2c). The hybridization was performed according to the manufacturer’s instruction and the cells were counterstained with DAPI II (Abbott Molecular) after the hybridization. FISH results were analyzed and documented using CytoVision system (Leica Microsystems).

## Results

### Array CGH findings

Array CGH analysis of the proband revealed a 5.6 Mb interstitial deletion within the 6p25.1p24.3 region: arr[hg19] 6p25.1p24.3(4,745,144-10,384,769)x1 (Fig. 2a). This deleted region carries 17 annotated genes in the database of online Mendelian inheritance in man (OMIM) (*CDYL*, *RPP40*, *LYRM4*, *FARS2*, *NRN1*, *F13A1*, *LY86*, *RREB1*, *SSR1*, *CAGE1*, *DSP*, *BMP6*, *BLOC1S5*, *EEF1E1*, *SLC35B3*, *HULC* and *OFCC1*) and 10 other genes (*PPP1R3G*, *LY86-AS1*, *SNAPC5P1*, *CNN3P1, BTF3P7, RIOK1, SNRNP48, TXNDC5, BLOC1S5-TXNDC5* and *PIP5K1P1*).

The mother showed an apparently mosaic loss of the same region seen in the proband (Fig. 2a). The father was normal, in particular without detectable copy changes involving the 6p region (data not shown).

Due to the mother’s carrier status, the proband’s brother was also tested per the family’s request. Surprisingly, instead of the deletion, an interstitial duplication of the same region was detected: arr[hg19] 6p25.1p24.3(4,745,144-10,384,769)x3 (Fig. 2a). This result implied that the duplication might also be derived from the maternal genomic changes that could be more complicated than a simple mosaic deletion.

### Chromosome and FISH findings

To further evaluate the unusual array findings, conventional cytogenetic analyses of the siblings and their mother were performed. Indeed, the mother actually has three different cell populations. Of a total of 50 metaphase cells examined, 36 (72.0 %) showed the 6p deletion, 3 (6.0 %) showed the 6p duplication, and the remaining 11 (22.0 %) were normal. Her karyotype is: 46,XX,del(6) (p24p25.1) [36]/46,XX,dup(6) (p24p25.1) [3]/46,XX [11] (Fig. 2b).

The chromosome results were confirmed by both metaphase and interphase FISH studies using two BAC probes located at the proximal and distal ends of the deletion/duplication region, respectively, including RP11-339A7 (6p24.3, labeled with green fluorochrome) and RP3-520B18 (6p25.1, labeled with red fluorochrome) (Fig. 2c). Consistent with the chromosomal findings, all three cell populations were detected in the mother; deletion, duplication and normal signal patterns were detected in approximately 70.0 % (350/500), 8.4 % (42/500) and 21.6 % (108/500) of the interphase cells examined, respectively (Fig. 2d). The metaphase FISH further confirmed the deletion/duplication and provided no evidence of other rearrangements involving the 6p25.1p24.3 region (Fig. 2c). The deletion and duplication in the siblings were also confirmed by FISH (data not shown). These findings demonstrated that the proband and her brother apparently inherited the deletion and duplication from their carrier mother, respectively.

## Discussion

**Fig. 3:**
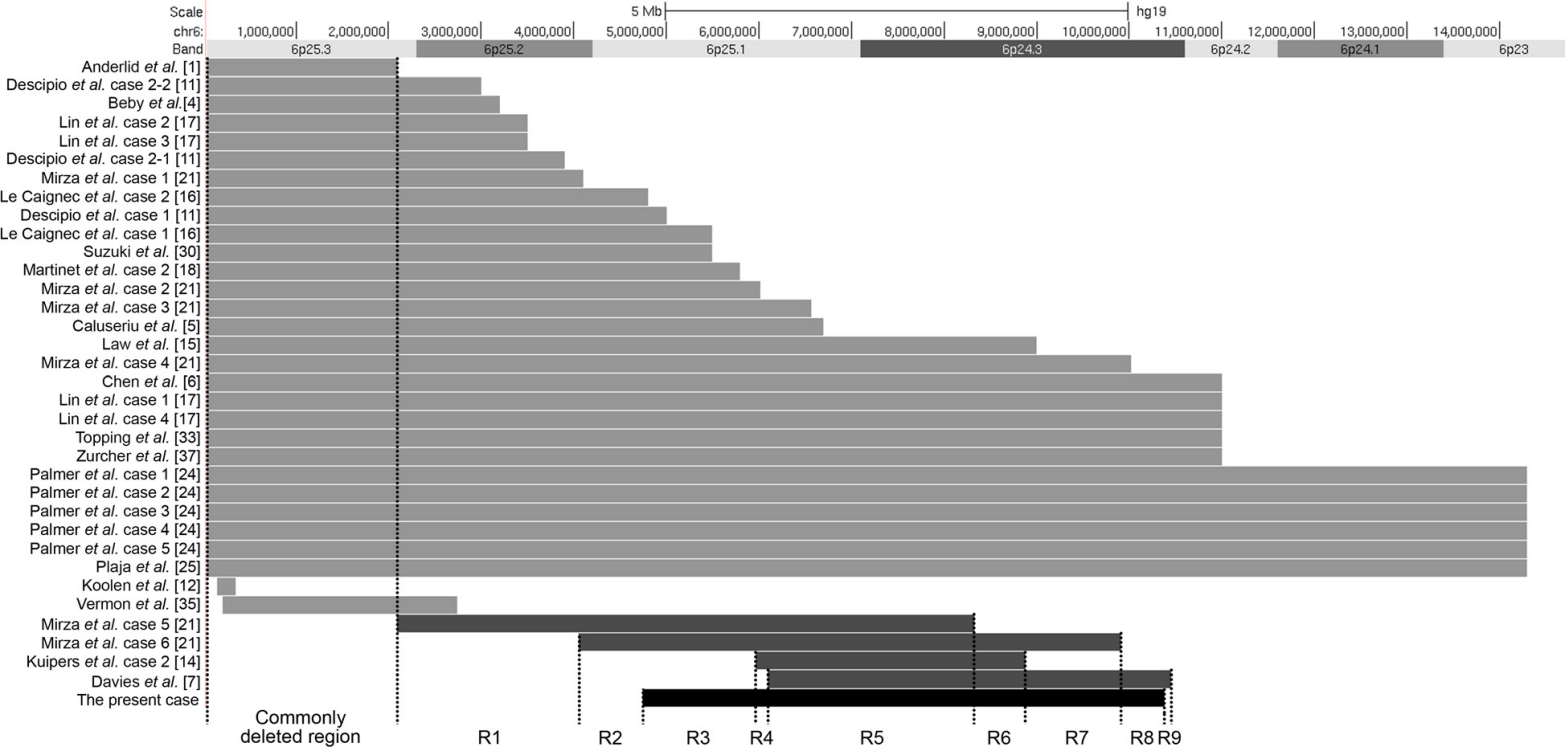
Reported deletions involving the 6pter-p23 region. Light gray bars represent the deletions involving the terminal 2.1 Mb region; dark gray bars represent the interstitial deletions without the involvement of the terminal 2.1 Mb region; black bar represents the present case of this study. The breakpoints of the five interstitial deletions divide the 6p25.2p24.3 into nine sub-regions, R1-R9. The breakpoint coordinates of Mirza *et al*. case 5 and case 6 and Davies *et al*. are estimated based on FISH data [7, 21]

The vast majority of deletions seen in the chromosome 6pter-p24 deletion syndrome are terminal deletions. The smallest reported terminal deletion was approximately 2.1 Mb (Fig. 3). This region was suggested to be responsible for some major features of the syndrome [1, 10]. However, the phenotypic contributions of other deleted 6p regions remain unknown. Isolated interstitial deletions within 6p25p24 without involvement of the terminal 2.1 Mb region are uncommon. To our knowledge, only five such deletions, including the one presented in this study, have been reported (Fig. 3) [7, 14, 21]. Individuals with these deletions share many common phenotypic features of the 6pter-p24 deletion syndrome (Table 1, also see the review of DeScipio [10]). Based on the breakpoints of the five overlapping interstitial deletions, nine sub-regions (R1-R9) can be defined along with annotated OMIM genes (Fig. 3 and Table 2). The region R5 (6,100,000-8,330,000 bp) that carries nine OMIM genes appears to be the consensus region of the deletions. Three genes in this region, including *RREB1*, *DSP* and *BMP6*, are of particular interest. The *RREB1* gene encodes a zinc finger transcription factor (RREB1 protein) that binds to RAS-responsive elements (RREs). It has been suggested that RREB1 is possibly involved in RAS/RAF-mediated cell differentiation and augments the RAS/RAF-mediated transcriptional response [31]. The RAS/RAF/MEK/ERK-signal transduction pathway is known to be associated with Noonan syndrome (OMIM #163950), Costello syndrome (OMIM #218040) and Cardio-Facial-Cutaneous syndrome (OMIM# 115150) [27, 32]. These syndromes share some of the phenotypic features of the 6p25.1p24.3 deletions, such as cardiac abnormalities, craniofacial dysmorphism and hemangioma [14, 27, 32]. Therefore, we speculate that deletion of the *RREB1* gene may underlie some, if not all, of these phenotypes through the RAS/RAF signal pathway. The *DSP* gene encodes desmoplakin protein that is an obligate component of functional desmosomes (the intercellular junctions that tightly link adjacent cells). Desmoplakin haploinsufficiency was suggested to be associated with keratosis palmoplantaris striata II (PPKS2; OMIM #612908), and recessive mutations in the *DSP* gene have been associated with skin fragility/woolly hair syndrome (OMIM #607655) and dilated cardiomyopathy (OMIM #605676) [2, 23]. Deletion of *DSP* could be related to abnormal findings of heart and hair. The *BMP6* gene encodes a bone morphogenetic protein 6. Kugimiya *et al.* and Meynard *et al.* reported that Bmp6 knockout mice had a delay in ossification, which was strictly confined to sternum [13, 19]. Thus, deletion of the *BMP6* gene could be the potential cause of pectus excavatum that was observed in four of the five individuals with the 6p25.1p24.3 deletions.

**Table 1:** Phenotypes associated with reported interstitial deletions involving 6p25.1p24.3

	Present case	Mirza *et al.* case 5 [21]	Mirza *et al.* case 6 [21]	Davies *et al.* [7]	Kuipers *et al*. case 2 [14]
Deleted region	4,745,144-10,384,769	2,090,000-8,330,000^*^	4,050,000-9,910,000^*^	6,100,000-10,450,000^*^	5,968,242-8,882,919
Size (Mb)	5.64	5.26	5.86	4.35	2.91
Age at first examination	2y2m	6 m	1y1m	1y11m	4y
Sex	Female	Female	Male	Male	Male
Growth delay	Mild				
Motor development delay		Moderate			Mild
Speech delay	Mild				
Hypotonia		+			+
Cardiac anomalies	PFO		PDA/ASD	ASD/VSD	
Conductive hearing loss	+				
Craniofacial dysmorphism					
Prominent forehead					+
Low-set ears		+			+
Posteriorly rotated ears		+			
Synophrys	+				
Hypertelorism	+	+	+	+	
Short palpebral fissures				+	
Blue sclera		+	+		
Microphthalmia with corneal clouding				+	
Broad nasal bridge	+	+			+
Flat nose			+	+	
Nasal tip			Bulbous		Full
Short philtrum	+				+
Highly arched palate		+			
Bilateral cleft lip			+		
Downturned corners of the mouth	+				
Micrognathia/retrognathia				+	+
Widely spaced and late erupting teeth	+				
Extremities					
Long fingers		+			+
Clinodactyly	+				+
Overlapping toes	+		+		
Mild joint hypermobility		+			+
Talipes				valgus	
Short neck		+			
Pectus excavatum	+	+	+	+	
Hemangioma	+ +	+			+
Umbilical hernia	+	+	+		
Hair abnormalities					+

Note: + feature present, ^*^the breakpoints are estimated based on FISH data [7, 21]; *PFO* patent foramen ovale, *PDA* patent ductus arteriosis, *ASD* atrial septal defect, *VSD* ventricular septal defect

**Table 2:** Nine regions defined by the breakpoints of reported interstitial deletions with annotated OMIM genes and potential phenotype map

Region	Start (bp)	End (bp)	Cases involved^*^	Reported phenotypes^**^	OMIM genes
R1	2090000	4050000	1	Highly arched palate, short neck, posteriorly rotated ears	*GMDS, WRNIP1, SERPINB1, SERPINB9, SERPINB6, NQO2, RIPK1, BPHL, TUBB2A, TUBB2B, SLC22A23, FAM50B, PRPF4B*
R2	4050001	4745144	1,2	Blue sclera	*CDYL*
R3	4745145	5968242	P,1,2	Umbilical hernia	*CDYL, RPP40, LYRM4, FARS2*
R4	5968243	6100000	P, 1, 2, 4	Motor development delay^***^, broad nasal bridge	*NRN1,*
R5	6100001	8330000	P, 1, 2, 3, 4	Pectus excavatum^***^, hemangioma^***^, cardiac anomalies^***^, hypotonia, hypertelorism^***^, low set ears^***^, hair abnormalities^***^, long fingers, mild joint hypermobility,	*F13A1, LY86, RREB1, SSR1, CAGE1, DSP, BMP6, BLOC1S5, EEF1E1*
R6	8330001	8882919	P, 2, 3, 4	Micrognathia/retrognathia	*SLC35B3, HULC*
R7	8882920	9910000	P, 2, 3	Cardiac anomalies, flat nose, bilateral cleft lip^***^, overlapping toes	*OFCC1*
R8	9910001	10384769	P, 3		
R9	10384770	10450000	3	Eye anomalies^***^	*TFAP2A*

^*^P: the present case; 1 and 2: Mirza *et al.* case 5 and case 6; 3: Davies *et al.*; 4: Kuipers *et al.* case 2 [7, 14, 21]; ^**^phenotypes were mapped by co-existence with the deletion region; ^***^phenotypes were also mapped by association with the known functions of the gene(s) in the deletion region

In addition, the region R4 contains the *NRN1* gene. The gene’s homologue in mouse encodes neuritin 1 protein that is involved in synaptic plasticity during the brain development possibly through controlling neuronal migration [22, 36]. This implies that deletion of *NRN1* may contribute to motor developmental delay. The region R7 contains the *OFCC1* gene that has been associated with isolated cleft lip with or without cleft palate (OFC1; OMIM %119530) [8]. One of the three cases with deletion of the *OFCC1* gene showed bilateral cleft lips (Table 1, Fig. 3), supporting the association. The region R9 contains the *TFAP2A* gene, of which haploinsufficiency may be associated with eye anomalies [7, 20]. Consistent with this, the case 3 showed microphthalmia with corneal clouding and it was the only case carried deletion of the region R9 including the *TFAP2A* gene (Table 2).

Based on the co-existence of phenotype/deletion and reported functions of the genes located in each deletion sub-region, some deletion-related phenotypes could be potentially mapped to the specific deletion sub-regions (Table 2). Such a phenotypic map, although it needs to be further verified, is important for understanding the genomic pathogenesis of the deletions. The phenotypes may not always manifest consistently together with the expected deletion regions. This could be due to incomplete penetrance/expressivity. Also, some phenotypes might not be examined or apparent at the time of reporting. Some shared phenotypes between the individuals with and without the 2.1 Mb terminal deletion could be due to the overlapping deletion regions of the two groups (Fig. 3). It is also possible that some of the phenotypes could be caused by different genomic mechanisms or by multiple genomic alterations.

To our knowledge, only two duplications involving the distal part of 6p have been reported with abnormal clinical findings [29, 34]. However, one of them did not overlap with, and the other was 4.5 Mb (80 %) larger in size (with many additional genes involved) than the present duplication. We also searched DGV (database of genomic variants, http://dgv.tcag.ca/), ClinGen (database of submitted clinical array testing results, http://clinicalgenome.org/) and DECIPHER (database of genomic variation and phenotype in humans using Ensembl resources, https://decipher.sanger.ac.uk/) databases, but no similar duplications were found. The DECIPHER database lists 10 much smaller duplications (0.09 – 2.4 MB) within the 6p region. However, three of them involve multiple additional genomic abnormalities and another three are listed without phenotypes. The phenotypic features associated with the remaining four duplications are not described in detail; in particular, no consistent specific phenotypes are listed. Therefore, the phenotypic features associated with those small duplications could not be determined. In contrast, the proband’s brother who carries a duplication within 6p25.1p24.3 in this study is phenotypically normal at the age of 5 years, suggesting that copy gain of this particular region is likely benign or triploinsensitive. It is noteworthy that approximately 70 % of the blood cells of the proband’s mother carry the deletion, but she apparently does not have most of the phenotypic features seen in the proband except for a mild cardiac anomaly. This implies that dosage compensation from the cells with the duplication might play a role in reducing the phenotypic effect of the deletion, although this might also be due to tissue-specific mosaicism that was not tested in this study. Nevertheless, the findings suggest that the level of phenotypic expression of the 6p25.1p24.3 deletion seems to be related with the overall level of mosaicism of the deletion.

**Fig. 4:**
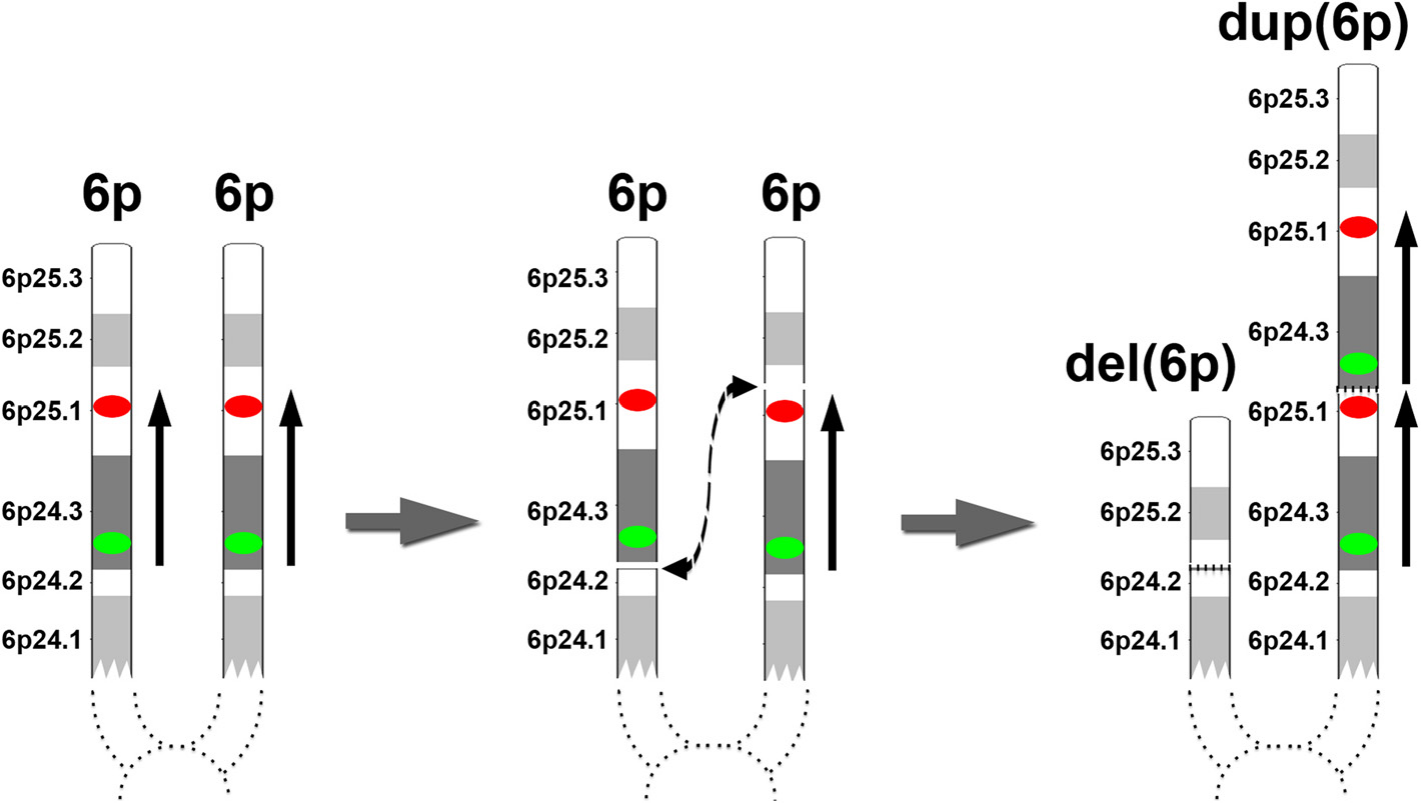
Unequal sister chromatid exchange involving the 6p25.1p24.3 region

The mosaic reciprocal deletion and duplication in the mother were most likely derived from a mitotic unequal sister chromatid exchange (Fig. 4). When this occurs between two non-allelic loci in a cell during mitosis, it gives rise to two cell populations with a deletion and a reciprocal duplication, respectively, along with an uninvolved normal cell population in an individual. This mitotic non-allelic recombination most likely occurred at an early stage during the maternal embryogenesis, since the percentage of the deletion cell population appeared to be high and the mosaicism was present in multiple tissues, such as blood and gonadal tissues. The mechanism for this unequal sister chromatid exchange is unknown. No segmental duplications, except for a 277-bp *Alu* repeat, were found in the proximity of the breakpoints in our search using the UCSC genome browser. However, whether this repeat was involved in the unequal exchange needs to be further determined, although *Alu* repeats have been reported to participate in some homologous recombination events [9, 26].

Our study clearly demonstrated the necessity to verify abnormal cytogenomic array findings with cytogenetic methods, including chromosomal analysis when the changes are >3 Mb and metaphase FISH when the changes are <3 Mb. Cytogenetic methods do not only confirm the array findings, but also allow identification of causal structural rearrangements of the array findings and multiple cell populations, as seen in this study, which would otherwise be missed. Such additional information is critical to understand the genomic pathogenesis, to make appropriate decision for clinical management and to provide appropriate counseling to the patient and his/her family.

## Conclusions

Our study demonstrated that an interstitial deletion within 6p25.1p24.3 and its reciprocal duplication could occur through mitotic unequal sister chromatid exchange. Both deletion and duplication may be passed independently to the offspring from a parental carrier if his/her gonadal system is affected. Cytogenomic array may detect the deletion or duplication in an individual, but may not detect both in a mosaic carrier. Therefore, the array findings could be misleading in such a mosaic situation. Chromosome and FISH analyses are appropriate methods to identify structural and copy number changes, as well as different cell populations. So, it is essential to verify abnormal cytogenomic array findings with chromosomal and FISH analyses when they are available. In review of other reported interstitial deletions within 6p25.1p24, nine deletion sub-regions with annotated OMIM genes, including a 2.3 Mb consensus region, are defined. Based on the known functions of the annotated genes and the phenotype/deletion co-existence, some phenotypes associated with the deletions can be potentially mapped to specific deletion regions. While the deletion in the proband of this study results in phenotypes reportedly associated with the chromosome 6pter-p24 deletion syndrome due to haploinsufficiency of the region, its reciprocal duplication appears not to have significant phenotypic effect, suggesting possible triploinsensitivity of the same region. In addition, the cells with the duplication may compensate the phenotypic effect of the cells with the deletion in the same individual as implied by the maternal karyotype and her mild phenotype.

## Consent

Written informed consent was obtained from the proband’s mother for publication of this Case report and any accompanying images. A copy of the written consent is available for review by the Editor of this journal.

## Abbreviations

aCGH: Array comparative genomic hybridization
BAC: Bacteria artificial chromosome
DGV: Database of genomic variants
DECIPHER: Database of genomic variation and phenotype in humans using Ensembl resources
FISH: Fluorescence in situ hybridization
ISCN: International system for human cytogenetic nomenclature
OMIM: Online Mendelian inheritance in man
